# Association of apical rocking with super-response to cardiac resynchronisation therapy

**DOI:** 10.1007/s12471-015-0768-4

**Published:** 2015-12-09

**Authors:** A. Ghani, P.P.H.M. Delnoy, J.J.J. Smit, J.P. Ottervanger, A.R. Ramdat Misier, A. Adiyaman, A. Elvan

**Affiliations:** Department of Cardiology, Isala, Dr. Van Heesweg 2, 8025 AB Zwolle, The Netherlands

**Keywords:** Super-response, Cardiac resynchronisation therapy, Apical rocking, Prognosis

## Abstract

**Background:**

Super-responders to cardiac resynchronisation therapy (CRT) show an exceptional improvement in left ventricular ejection fraction (LVEF). Previous studies showed that apical rocking was independently associated with echocardiographic response to CRT. However, little is known about the association between apical rocking and super-response to CRT.

**Objectives:**

To determine the independent association of LV apical rocking with super-response to CRT in a large cohort.

**Methods:**

A cohort of 297 consecutive heart failure patients treated with primary indication for CRT-D were included in an observational registry. Apical rocking was defined as motion of the left ventricular (LV) apical myocardium perpendicular to the LV long axis. ‘Super-response’ was defined by the top quartile of LVEF response based on change from baseline to follow-up echocardiogram. Best-subset regression analysis identified predictors of LVEF super-response to CRT.

**Results:**

Apical rocking was present in 45 % of patients. Super-responders had an absolute mean LVEF increase of 27 % (LVEF 22.0 % ± 5.7 at baseline and 49.0 % ± 7.5 at follow-up). Apical rocking was significantly more common in super-responders compared with non-super-responders (76 and 34 %, *P* < 0.001). In univariate analysis, female gender (OR 2.39, 95 % CI 1.38–4.11), lower LVEF at baseline (OR 0.91 95 % CI 0.87–0.95), non-ischaemic aetiology (OR 4.15, 95 % CI 2.33–7.39) and apical rocking (OR 6.19, 95 % CI 3.40–11.25) were associated with super-response. In multivariate analysis, apical rocking was still strongly associated with super-response (OR 5.82, 95 % CI 2.68–12.61). Super-responders showed an excellent clinical prognosis with a very low incidence of heart failure admission, cardiac mortality and appropriate ICD therapy.

**Conclusion:**

Apical rocking is independently associated with super-response to CRT.

**Electronic supplementary material:**

The online version of this article (doi:10.1007/s12471-015-0768-4) contains supplementary material, which is available to authorized users.

## Introduction

Cardiac resynchronisation therapy with a defibrillator (CRT-D) has proven to improve heart failure morbidity, quality of life, and survival in those with reduced left ventricular ejection fraction (LVEF), advanced heart failure symptoms, and increased QRS duration [1–5]. Recent studies have indicated super-response in a proportion of patients treated with CRT [6, 7]. Identifying potential super-responders to CRT is an important issue because of their excellent prognosis. Previous studies attempted to find easily identifiable clinical factors to predict super-response to CRT. Female gender, body mass index (BMI) < 30 kg/m^2^, left bundle branch block (LBBB), QRS duration > 150 ms, smaller left ventricular (LV) and left atrial (LA) dimensions, shorter duration of heart failure symptoms, and non-ischaemic cardiomyopathy were factors associated with super-response to CRT [8–11], albeit with a relatively weak relation. There is an obvious need for a stronger predictor for these patients. Apical rocking is an easily measured echocardiographic parameter, and has been introduced as a promising predictor of CRT [12–14]. However, to our knowledge, there are no data on the value of apical rocking as a predictor of super-response to CRT. Therefore, the aim of the current study was to assess the value of apical rocking as an independent predictor of super-response to CRT in a large cohort of patients treated with CRT-D.

## Methods

### Selection of patients

**Fig. 1:**
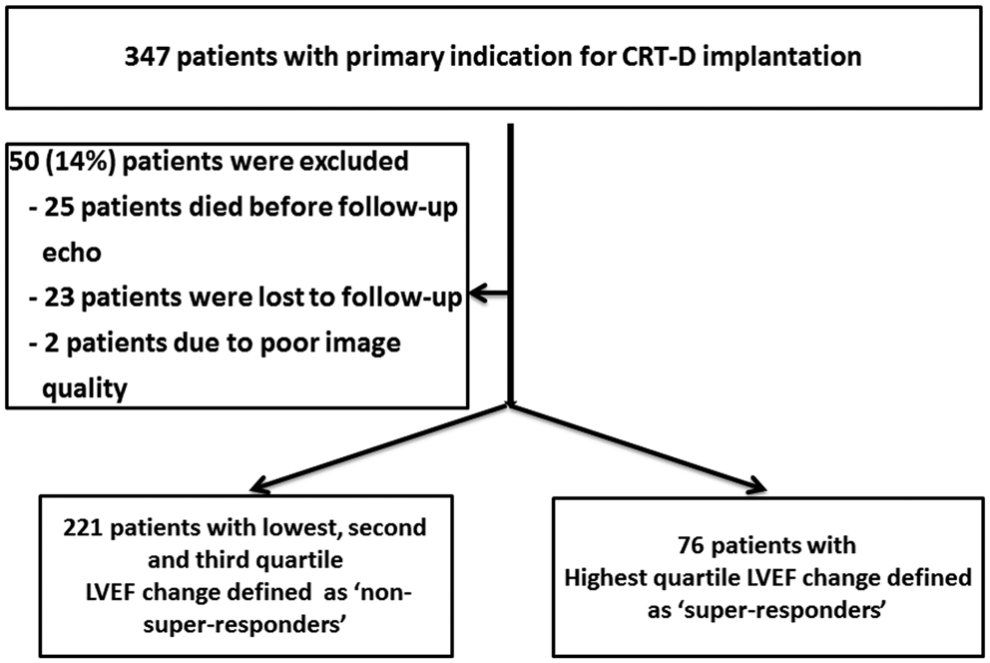
Flowchart of study population

Between 2005 and 2009, patients with a primary indication for CRT-D were included in a prospective registry. This study is an extension of our previous study [14] with a larger number of patients and longer duration of follow-up. This prospective registry has been approved by the Institutional Review Board. Exclusion criteria were: (1) patients with CRT pacemaker (CRT-P), (2) pre-implantation LVEF > 35 % according to echocardiographic data, (3) patients with a history of recent myocardial infarction or coronary artery bypass graft (< 3 months). Indication for CRT-D implantation was determined according to the guidelines at the time of implantation. In all patients, LVEF was ≤ 35 % and QRS duration was > 120 ms with LBBB, RBBB or non-specific intraventricular conduction disorders (IVCD). Conventional criteria for LBBB were used, which include QRS duration ≥ 120 ms, QS or rS in lead V1, and a monophasic R wave in leads V6 and I without Q waves. Heart failure was diagnosed according to the European Society of Cardiology guidelines. Medical therapy was optimised to reach the highest tolerated dosages of angiotensin-converting enzyme inhibitors and beta-blockers. To be included in the final analysis, patients were required to have an echocardiographic examination before CRT-D implantation and during follow-up. Based on these criteria, a total of 297 patients were eligible for this study as depicted in Fig. 1. LVEF assessment was performed in all patients at baseline and follow-up. LVEF was calculated using the Simpson’s technique [15].

### Device implantation

CRT devices from all major manufacturers (Medtronic, St Jude Medical, Boston Scientific, Biotronik and Sorin Group) were implanted. The majority of coronary sinus leads were bipolar. After implantation, tailored device programming was performed before discharge with 3 consecutive zones in the large majority of patients. A monitor zone between 170 and 200 bpm, fast ventricular tachycardia (VT) zone between 200 and 230 bpm and ventricular fibrillation (VF) zone > 230 bpm. In the fast VT zone, arrhythmias were initially attempted to be terminated by 2 bursts and 1 ramp followed by defibrillator shocks if the arrhythmia continued. Routine follow-up visits were scheduled at 2 months post implant, and every 6 months thereafter. During follow-up, ICD printouts were checked, ICD treatments were registered and intracardiac electrograms were classified by a dedicated device cardiologist. Appropriate ICD therapy (anti-tachycardia pacing and shocks) was defined as ICD therapy delivered in response to sustained ventricular tachycardia or ventricular fibrillation.

### Visual assessment of LV-apical rocking

Apical rocking was defined as a short initial septal contraction which results in short inward motion of the septum pulling the apex to the septum, and then the delayed activation of the lateral wall which pulls the apex laterally during the ejection time while stretching of the septum takes place. The presence of apical rocking was visually assessed in the 4-chamber apical view by three experienced cardiologists who were blinded to the medical history, LVEF measurements, and clinical outcome of the study population. The presence of apical rocking in some patients was difficult to assess adequately, in these cases we followed the democratic majority. Interobserver and intraobserver variability was expressed as kappa coefficients. Values higher than 0.8 are considered to be excellent, values between 0.6 and 0.8 as good, values between 0.4 and 0.6 as moderate, and values below 0.4 as poor agreement.

### Endpoint

Patients with paired echocardiograms were divided into quartiles of LVEF response to CRT based on change from baseline to follow-up echocardiograms. Two groups based on response to CRT were defined and labelled as ‘super-responders’ and ‘non-super-responders’ [11]. Super-response to CRT was defined by the highest quartile of LVEF change (*n* = 76) and non-super-response by the lowest, second and third quartiles of LVEF change (*n* = 221).

### Statistical analysis

Statistical analysis was performed using SPSS statistical software (IBM Corp. Released 2011. IBM SPSS Statistics for Windows, Version 22.0. Armonk, NY: IBM Corp). Continuous variables were expressed as mean ± SD and significance of differences between independent groups were calculated using the nonparametric Mann-Whitney U test. Categorical variables were presented as number and percentages, and the significance of differences between groups was calculated using the chi-squared test or Fisher’s exact test as appropriate. Logistic regression analysis was performed to assess the univariable and multivariable predictors super-response versus non-super-response. We computed the sensitivity and specificity of apical rocking in predicting super-response. The probability of clinical outcomes was plotted using Kaplan-Meier estimates, and groups were compared using log-rank tests. *P*-values < 0.05 were considered statistically significant in all analyses.

## Results

### Baseline characteristics

Initially 347 patients with prophylactic CRT-D indication were registered in our hospital database. Paired echocardiograms from both baseline and follow-up were not available in 50 (14 %) patients. Of these patients, 25 (50 %) patients died before follow-up echocardiograms were performed. Therefore, these patients were excluded from analysis. The final study population consisted of 297 patients (Fig. 1). Echocardiographic follow-up was performed at median 2.1 years (IQR 1.4–3.2) after device implantation. General characteristics of the study population are summarised in Table 1 and 2. The median age was 68.7 (IQR 58–78) years with 30 % females. Median LVEF was 24.8 % (IQR 20–30 %), LBBB was present in 81 %, the median QRS duration was 160 ms (IQR 132–182 ms) and 49 % of patients had non-ischaemic aetiology. The coronary sinus leads were positioned in the lateral, posterolateral or posterior region in 83 % and anterolateral or anterior in 8 % of patients. Furthermore, 9 % of LV leads were positioned epicardially during open heart surgery prior to CRT-D implantation. A total of 76 patients were classified as super-responders. A higher proportion of women, patients with non-ischaemic aetiology and with baseline apical rocking were among the super-responders to CRT. At follow-up, the median LVEF was 37 % (IQR 20–32 %) in all patients, 38 % (IQR 30–43 %) in non-super-responders and 49 % (IQR 45–52 %) in super-responders (*P* < 0.001) (Table 3).

**Table 1:** General characteristics of study population by apical rocking

	With apical rocking *N* = 134	Without apical rocking *N* = 163	*p*-value
**Age (years) median (IQR)**	67.3 (58.7–72.2)	70.2 (63.5–75.2)	0.001
**Female**	41 %	22 %	< 0.001
**LVEF (%) median (IQR)**	25.0 (20.0–30.0)	24.7 (20.0–30.0)	0.929
**Sinus rhythm**	81 %	71 %	0.038
**QRS duration (ms) median (IQR)**	162 (147–180)	150 (128–170)	< 0.001
**LBBB**	91 %	73 %	< 0.001
**RBBB**	3 %	9 %	0.055
**IVCD**	6 %	18 %	0.003
**NYHA functional class median (IQR)**	3.0 (2.0–3.0)	3.0 (2.0–3.0)	0.637
**Non-ischaemic aetiology**	71 %	31 %	< 0.001
**Diuretics**	77 %	86 %	0.044
**Βeta-blocker**	85 %	80 %	0.290
**AT-II receptor blockers**	48 %	40 %	0.137
**ACE-inhibitors**	77 %	75 %	0.782
**Spironolactone**	42 %	45 %	0.611

*ACE* angiotensin-converting enzyme, *AT-II* angiotensin II, *IQR* interquartile range, *IVCD* intraventricular conduction disorders, *LVEF* left ventricular ejection fraction, *LBBB* left bundle branch block, *RBBB* right bundle branch block, *NYHA* New York Heart Association.

**Table 2:** General characteristics of study population by responder category

	Non-super-responder *N* = 221	Super-responder *N* = 76	*p*-value
**Age (years) median (IQR)**	69.3 (61.5–74.3)	67.6 (59.4–72.9)	0.262
**Female**	25 %	45 %	0.002
**LVEF (%) median (IQR)**	25.0 (21.0–30.0)	22.0 (18.0–25.5)	< 0.001
**Sinus rhythm**	75 %	76 %	0.821
**QRS duration (ms) median (IQR)**	160 (135–173)	160 (132–182)	0.238
**LBBB**	80 %	86 %	0.281
**RBBB**	7 %	5 %	0.770
**IVCD**	13 %	9 %	0.432
**NYHA functional class median (IQR)**	3.0 (2.0–3.0)	3.0 (2.0–3.0)	0.502
**Non-ischaemic aetiology**	40 %	74 %	< 0.001
**Diuretics**	83 %	79 %	0.452
**Βeta-blocker**	81 %	87 %	0.216
**AT-II receptor blockers**	42 %	47 %	0.383
**ACE-inhibitors**	76 %	74 %	0.625
**Spironolactone**	46 %	38 %	0.253
**Presence of apical rocking**	34 %	76 %	< 0.001

*ACE* angiotensin-converting enzyme, *AT-II* angiotensin II, *IQR* interquartile range, *IVCD* intraventricular conduction disorders, *LVEF* left ventricular ejection fraction, *LBBB* left bundle branch block, *RBBB* right bundle branch block, *NYHA* New York Heart Association.

**Table 3:** Left ventricular ejection fraction (LVEF) changes and presence of apical rocking between different groups at baseline and follow-up

	Non-super-responders (*N* = 221)		Super-responder (*N* = 76)	*p*-value
	Non-responders (*N* = 73)	Responders (*N* = 148)		
**LVEF (%) at baseline median(IQR)**	25.0 (21.0–29.0)	25.0 (21.0–31.5)	22.0 (18.0–25.5)	< 0.001
**LVEF (%) during follow-up median(IQR)**	24.0 (20.0–28.0)	38.0 (30.0–43.0)	49.0 (45.0–52.0)	< 0.001
**LVESV decrease (ml) mean ± SD**	4.3 ± 35.8	31.4 ± 37.6	6.5 ± 61.6	< 0.001
**Presence of apical rocking**	15 %	44 %	76 %	< 0.001

The groups were defined as: Super-responders: the highest quartile of LVEF change; responders the second and third quartiles of LVEF change; and non-responders the lowest quartile of LVEF change.

*LVESV* left ventricular end-systolic volume.

### Interobserver and intraobserver variability

To quantify the interobserver and intraobserver variability for assessment of apical rocking, 140 (47 %) patients were reviewed by 3 cardiologists. The interobserver variability kappa was 0.85 and intraobserver variability kappa was 0.90 between the 3 cardiologists.

### Long-term outcome of super-responders

**Fig. 2:**
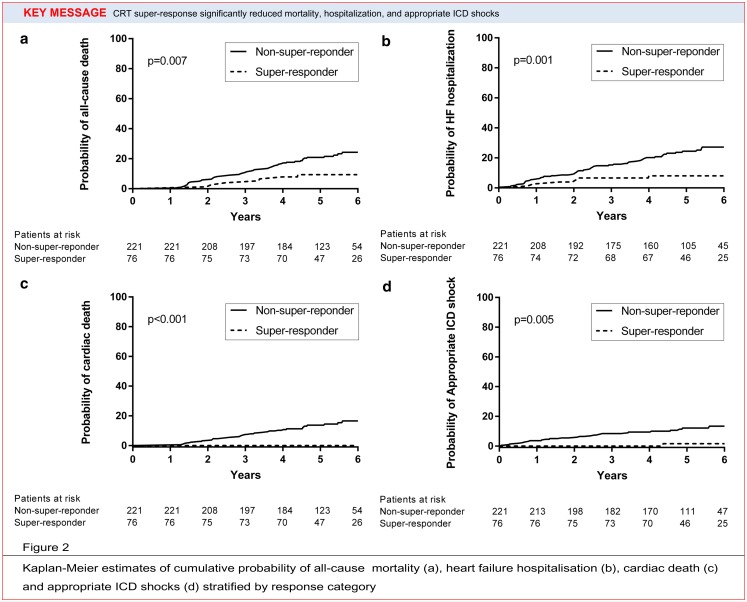
Kaplan-Meier estimates of cumulative probability of all-cause mortality (**a**), heart failure hospitalisation (**b**), cardiac death (**c**) and appropriate ICD shocks (**d**) stratified by response category

Patients were followed for a median of 5.2 years (IQR 4.4–6.2). During this period 63 (21 %) patients died (all-cause mortality). The mode of death was cardiac in 33 patients (11 %) and non-cardiac or unknown in 30 patients (10 %). All-cause mortality in super-responders was significantly lower compared with non-super-responders (11 vs 25 %, *P* = 0.008, Fig. 2a). None of the super-responders died from a cardiac cause, whereas a cumulative incidence of cardiac cause up to 20 % was observed in non-super-responders (Fig. 2c). During total follow-up, 21 % of patients were admitted to hospital due to worsening of heart failure. The rate of hospitalisation was significantly lower in super-responders compared with non-super-responders (8 vs 25 %, *P* = 0.001, Fig. 2b). Appropriate CRT-D shock was significantly lower in super-responders compared with non-super-responders (1 vs 12 %, *P* = 0.006, Fig. 2d). Inappropriate ICD shock did not differ between the super-responders and non-super-responders (10 vs 11 %, *P* = 0.977).

### Apical rocking and super-response to CRT

The presence of apical rocking between super-responders and non-super-responders to CRT was significantly different (76 and 34 % respectively, *P* < 0.001). Presence of apical rocking predicted super-response to CRT with a sensitivity of 76 % and a specificity of 66 %. The positive predictive value of apical rocking in predicting super-response was 44 % and the negative predictive value was 89 %, with an accuracy was 68 %. In univariate analysis, female gender (OR 2.39 95 %CI 1.38–4.11), LVEF at baseline (OR 0.91 95 %CI 0.87–0.95), non-ischaemic aetiology (OR 4.15 95 %CI 2.33–7.39) and apical rocking (OR 6.19 95 %CI 3.40–11.25) were associated with super-response to CRT. In multivariate analysis, female gender (OR 2.14 95 %CI 1.07–4.29), LVEF at baseline (OR 0.89 95 %CI 0.84–0.94) and apical rocking (OR 5.82 95 %CI 2.68–12.61) were associated with super-response to CRT after adjustment for age, LVEF baseline, QRS duration, LBBB vs non-LBBB, non-ischaemic aetiology and presence of apical rocking (Table 4).

**Table 4:** Multivariate association to CRT super-response

	Odds ratio	95 % CI	*P*-value
**Age (years)**	1.01	0.97–1.05	0.585
**Female**	2.14	1.07–4.29	0.032
**LVEF (%) baseline**	0.89	0.84–0.94	< 0.001
**QRS duration (per 10 ms)**	0.98	0.87–1.12	0.795
**LBBB vs non-LBBB**	0.91	0.33–2.49	0.858
**Non-ischaemic aetiology**	1.99	0.96–4.12	0.063
**Presence of apical rocking**	5.82	2.68–12.61	< 0.001

Adjusted for age, gender, LVEF, QRS width, LBBB/RBBB and ischaemic/non-ischaemic cardiomyopathy.

*LVEF* left ventricular ejection fraction, *LBBB* left bundle branch block, *RBBB* right bundle branch block.

## Discussion

The present study assessed the association of apical rocking with super-response to CRT in a large cohort of patients. Apical rocking was strongly associated with super-response. Furthermore, super-responders had a lower incidence of cardiac death, heart failure hospitalisation and appropriate ICD shocks.

Super-response is associated with decreased cumulative probability of heart failure or all-cause mortality and ICD therapy for ventricular tachycardia or ventricular fibrillation. Therefore, predicting super-response is important. Previous studies tried to find predictors of super-response. LBBB and smaller left atrial volume were previously identified as predictors of super-response [16, 17]. The MADIT-CRT trial [11] identified female gender, no prior myocardial infarction, QRS duration ≥ 150 ms, LBBB, BMI < 30 kg/m^2^ and smaller left atrial volume index as predictors of super-response.

The present study assessed several general characteristics and apical rocking as a echocardiographic parameter to predict the potential super-response, and identified lower baseline LVEF, female gender and apical rocking as predictors of super-response to CRT. Although the association of higher baseline LVEF with ‘normal CRT response’ has been established, in the current study lower baseline LVEF was associated with ‘super-response to CRT’. One of the recent trials [18] demonstrated that super-responders had lower baseline LVEF compared with non/modest responders (22.6 vs 25.8 %, *P* < 0.001). Our results are in line with this trial. However, in the MADIT-CRT trial [11] there were no significant differences in baseline LVEF between super-responders and non-super-responders. Apical rocking, as we recently published, predicted the response to CRT [14] and can be visualised in a standard echocardiographic four-chamber view. This is in contrast to several dyssynchrony indices, which require well-trained echocardiographers and special imaging software and techniques. A previous study compared a quantitative measurement with visual assessment of apical rocking and demonstrated a comparable accuracy in predicting CRT response [13]. Therefore, in this study we decided to use only visual assessment which can be assessed easily with a good interobserver and intraobserver variability. In 2007 Jansen et al. [19] described apical shuffle as an abnormal systolic septal-to-lateral motion of the left ventricle. Apical shuffle, has been shown to be predictive of LV reverse remodelling with sensitivity and specificity between 70 and 90 %. The investigators, however, did not describe the pathophysiological mechanism of apical shuffle. In recent years, the pathophysiological mechanism of apical rocking, defined as short-lived early septal motion of the apex and a predominantly lateral motion during ejection, has been described in 2 separate publications [12, 13]. Apical rocking is the same phenomenon as described by Jansen et al. [19], however, they called this abnormal movement of the apex ‘apical shuffle’. Septal rebound stretch (SRSsept) is another relatively new dyssynchrony parameter. Previous studies [20–22] demonstrated the strong association of SRSsept with CRT response. Septal rebound stretch reflects the amount of stretch in septum during systole and seems comparable with ’multiphasic septal motion’ which has been described by Jansen et al. [19]. However, in the current study we did not assess the predictive value of SRSsept on ‘super-response to CRT’ because we only had data on septal rebound stretch in a minority of patients*.* Our study, as far as we can ascertain, is the first to demonstrate the association between apical rocking and super-response. Although we predefined our LVEF response categories carefully, and found a strong association between apical rocking and super-response, we realise that our results should be confirmed in large multicentre trials. Although apical rocking has a strong association (OR 5.82, 95 % CI 2.68–12.61) with super-response as compared with patients without apical rocking, we emphasise that even in patients with apical rocking only 44 % are super-responders. This low positive predictive value of 44 % is dependent on the definition of super-response and low prevalence of super-response in our cohort. The absence of apical rocking is a strong predictor of non-super-response with a negative predictive value of 89 %. However, the absence of apical rocking was not our focus in the current study.

The response to CRT can change over time, particularly shortly after CRT. In our cohort echocardiographic examination after CRT implantation was performed after a mean of 2.1 years (IQR 1.4–3.2). The time from implantation to follow-up echocardiogram was comparable in both groups [in non-super-responders 2.1 years (IQR 1.4–3.3) and in super-responders 2.1 years (IQR 1.4–3.1), *p* = 0.80)]. So, we do not think that timing of echocardiography caused misclassification of super-responders.

### Long-term outcome in super-responders to CRT

The cumulative probability of all-cause mortality, heart failure hospitalisation, cardiac death and appropriate ICD therapy for VT or VF differed significantly across LVEF response categories at 6 years of follow-up, with improved event-free survival based on the magnitude of response (Fig. 2). In the current study we observed 11 % all-cause mortality, 8 % hospitalisation due to heart failure and 1 % appropriate ICD therapy in super-responders. None of super-responders died from cardiac causes. In MADIT-CRT [11] all-cause death occurred in 1.6 % and all-cause death or appropriate CRT-D therapy in 5.2 % of super-responders. However, in MADIT-CRT, follow-up was shorter (median 15 months). Another recent trial with 259 CRT patients and mean follow-up of 5.6 years showed a cardiovascular mortality of 1.5 % and all-cause mortality of 6 % in super-responders defined as LVEF > 50 % [23]. One of the largest trials with 92 super-responders (LVEF > 50 %) demonstrated that the survival rate was similar to that of the age- and sex-matched general population with appropriate shocks in 4.4 % of patients [24] during a mean follow-up of 5.7 ± 2.4 years. Given the good prognosis of super-responders which is demonstrated in previous studies, including the current study, we should be able to identify these patients and apical rocking may play an important role.

### Clinical implications

Identification of potential super-responders prior to implantation and during follow-up has several advantages. Super-responders have very good prognosis in terms of lower rate of heart failure hospitalisation and all-cause mortality. Furthermore, the incidence of cardiac death or appropriate ICD therapy is very low. These are important issues to discuss with patients prior to implantation. In super-responders, during follow-up when a device change is necessary due to battery depletion or dysfunction of a high voltage RV lead, downgrading from CRT-D to CRT-P can be discussed. Absence of apical rocking has a strong relation with ‘non-super-response’. It may therefore be used to identify the non-super-responders who may require more intensive monitoring during follow-up.

### Strengths and limitations

Both the large size of the study population and the long-term clinical follow-up are probably the major strengths of the current study. For the definition of ‘super-response’ we used the top quartile of LVEF response based on change from baseline to follow-up, exactly the same definition as in the MADIT-CRT trial [11], whereas other studies used an absolute LVEF > 50 % as cut-off for super-response. Changes in LVEF as a definition of super-response can be difficult to interpret. A patient can show both a decrease and an increase in LV end-diastolic and end-systolic volume, so the LVEF remains relatively unchanged. Therefore, non-response or response to CRT can be unnoticed. The follow-up echocardiographic examinations were performed at a median of 2.1 years (IQR 1.4–3.2), which means that all potential LV remodelling has taken place, as demonstrated in a previous study [5]. However, the majority of the studies performed the follow-up echocardiography at 6–12 months post-implantation. Furthermore, our data concern observations of a single centre, although with high experience in CRT. Our study focused on patients with available baseline and follow-up echocardiograms. Therefore, a proportion of patients (14 %) were excluded from the analysis. These patients included those who died before follow-up echo or were lost to follow-up because of referral to their own regional hospital. Another limitation of the current study is that the Kaplan-Meier graphs started immediately after the implantation whereas defining of response group by follow-up echocardiograms took place at a mean of 2.1 years. The current study population most closely resembles real life with inclusion of patients with atrial fibrillation. Visualisation of apical rocking was not negatively influenced by the inclusion of patients with atrial fibrillation. Suboptimal LV-lead placement or unfavourable pacemaker settings may, at least in part, have contributed to diminished improvement of LVEF and poorer outcome after CRT. In our population, no information is available on optimisation during follow-up.

## Conclusion

Apical rocking is independently associated with super-response to CRT. Apical rocking may therefore play an important role in identifying these patients, who seem to have a good long-term prognosis. Absence of apical rocking has a high negative predictive value for prediction of non-super-response.

### Sources of funding

None.

## Electronic supplementary material

(PPTX 13186 kb)
