# Supplementary figures and images for: Association of apical rocking with super-response to cardiac resynchronisation therapy
Source: Neth Heart J. 2015 Dec 9;24(1):39–46. doi: 10.1007/s12471-015-0768-4 (PMC4692828; doi:10.1007/s12471-015-0768-4)

## Slide 1
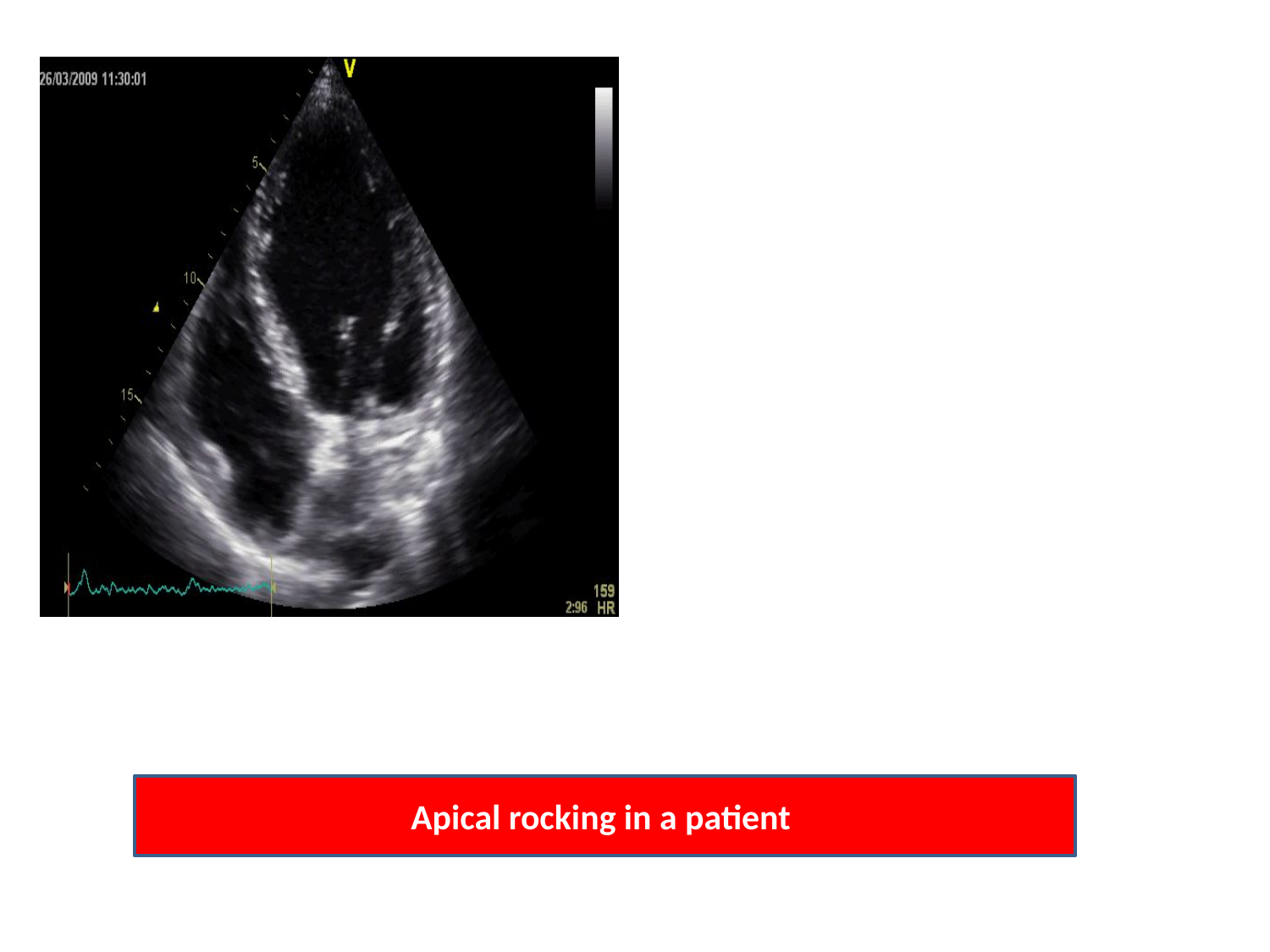

Apical rocking in a patient

Supplement: Supplementary file 1 — (PPTX 13186 kb) [file 12471_2015_768_MOESM1_ESM.pptx]
